# Congenital Portosystemic Shunt: Our Experience

**DOI:** 10.1155/2015/691618

**Published:** 2015-02-02

**Authors:** Tiziana Timpanaro, Stefano Passanisi, Alessandra Sauna, Claudia Trombatore, Monica Pennisi, Giuseppe Petrillo, Pierluigi Smilari, Filippo Greco

**Affiliations:** ^1^Unit of Clinical Pediatrics, Department of Medical and Pediatric Sciences, University of Catania, Via Santa Sofia, 95123 Catania, Italy; ^2^Radiodiagnostic and Oncological Radiotherapy Unit, University Hospital “Policlinico-Vittorio Emanuele”, Via Santa Sofia, 95123 Catania, Italy

## Abstract

*Introduction*. Congenital portosystemic venous malformations are rare abnormalities in which the portal blood drains into a systemic vein and which are characterized by extreme clinical variability. *Case Presentations*. The authors present two case reports of a congenital extrahepatic portosystemic shunt (Type II). In the first patient, apparently nonspecific symptoms, such as headache and fatigue, proved to be secondary to hypoglycemic episodes related to the presence of a portosystemic shunt, later confirmed on imaging. During portal vein angiography, endovascular embolization of the portocaval fistula achieved occlusion of the anomalous venous tract. In the second patient, affected by Down's syndrome, the diagnosis of a portosystemic malformation was made by routine ultrasonography, performed to rule out concurrent congenital anomalies. Because of the absence of symptoms, we chose to observe this patient. *Conclusions*. These two case reports demonstrate the clinical heterogeneity of this malformation and the need for a multidisciplinary approach. As part of a proper workup, clinical evaluation must always be followed by radiographic diagnosis.

## 1. Introduction

Congenital portosystemic shunts are rare vascular abnormalities in which the portal blood drains into a systemic vein. They are the result of embryogenetic alterations or the persistence of the fetal circulation elements, especially those related to the ductus venosus [1]. It is also associated with chromosomal abnormalities, especially Down's syndrome [2].

Anatomically congenital portosystemic venous shunts are classified into extra- and intrahepatic shunts [3]. To date, although these abnormalities are increasingly recognized due to the evolution and development of diagnostic imaging, the total number of cases described in the literature remains low. Of all published cases, 185 and 131 describe an extra- and intrahepatic portosystemic shunt, respectively [4].

Clinical presentation, especially in children, is extremely variable. In some cases, portosystemic malformations may remain asymptomatic, making the diagnosis difficult. In other cases, they may cause metabolic disorders and damage various organs and systems, such as the liver, central nervous system, and respiratory tract.

Imaging plays an important confirmatory role when the diagnosis is suspected and can clearly demonstrate the venous shunt, identify any associated malformations, and suggest the most appropriate management approach.

We present two patients who have been affected by congenital portosystemic shunts. Our two cases demonstrate how the malformation is characterized by heterogeneous clinical variability and can result in different therapeutic implications.

## 2. Case Report 1

Patient 1 was born from nonconsanguineous parents at the 37th week of gestation by spontaneous vaginal delivery with a birth weight of 2280 g (small for gestational age). At birth, he had low glucose levels and received an intravenous 10% glucose infusion with subsequent improvement in the following hours.

Since the age of six, he was regularly followed up by a pediatric neuropsychiatrist for attention hyperactivity disorder, difficulty with concentration, dyscalculia, dyslexia, and a “borderline” IQ.

At the age of 10, due to headache, severe fatigue, and daytime sleepiness he was admitted to our pediatric clinic for further investigation. His height was 149 cm (<90th percentile), his weight was 37.7 kg (25–50th percentile), and his head circumference was 53 cm (75th percentile).

On physical examination, he demonstrated an elongated facies, low-set ears, single palmar creases bilaterally, and hypoplasia of the hypothenar eminence. He also displayed alterations in his osteoarticular system, including arachnodactyly, scapular winging, an ankle valgus deformity, joint laxity, bilateral pes planus, and a hallux valgus deformity.

**Figure 1:**
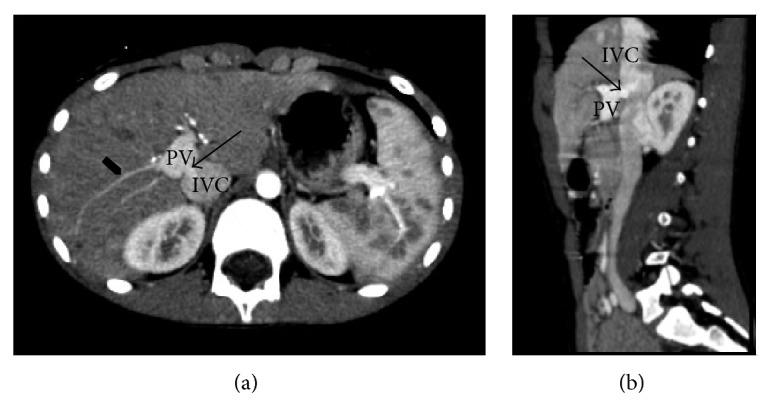
Contrast-enhanced CT images. Axial projection (a); sagittal-oblique projection (b). The arrows in (a) and (b) show a shunt between the posterior wall of the portal vein (PV), just before its intrahepatic hilar division, and the inferior cava vein (ICV); the intrahepatic portal branches appear reduced and filiform (arrowhead in (a)).

During the hospitalization, blood tests demonstrated hyperinsulinemia (36 *μ*U/mL; n.v 2.6–24.9) and mild hypoglycemia (49 mg/dL). He underwent oral glucose-tolerance testing, which revealed hyperinsulinemia at 60 minutes (395 *μ*U/mL, associated with a blood glucose value of 42 mg/dL). Testing while fasting was also performed but was stopped at 30 minutes due to hypoglycaemia and general malaise. Suspecting organic disease related to excessive insulin secretion, further diagnostic testing was performed. Ultrasonography (US) showed slight ectasia of the left renal vein coursing cranially and turbulent flow at the inferior vena cava (IVC) through the midline of the aortomesenteric branches. Contrast-enhanced magnetic resonance imaging (MRI) of the abdomen was performed, demonstrating hyperintense hepatic lesions on T1-weighted images and isointense lesions on T2-weighted images (diameters between 9 and 20 mm). No abnormalities of the venous circulation were identified at this point. The patient then underwent contrast enhanced CT (computed tomography) scan, which revealed similar hepatic nodular lesions, characterized by early enhancement with slow washout. These images also showed a close anatomical contiguity between the distal portal vein and the inferior vena cava, along with early uptake of contrast compatible with a portocaval shunt. An Abernethy malformation (Type II, side-to-side extrahepatic shunt) was confirmed (Figure 1).

In order to correctly classify the shunt, endovascular evaluation of the malformation was performed. The inferior vena cava was catheterized by percutaneous femoral access, and a venogram demonstrated the portocaval shunt. The caliber of the shunt was approximately 10 mm and the balloon occlusion test showed no intraportal pressure peaks. Therefore, embolization of the fistula was performed, achieving occlusion of the anomalous venous tract.

To date, clinical follow-up has been normal and the patient is in good health.

## 3. Case Report 2

Patient 2 was born from nonconsanguineous parents at the 36th week of gestation by spontaneous vaginal delivery with a birth weight of 1600 g (small for gestational age). The intrapartum diagnosis of Down's syndrome was made by amniocentesis. At the age of two, she underwent surgery for coarctation of the aorta and a patent ductus arteriosus. At the age of nine, she presented at our pediatric clinic for further diagnostic testing, given her underlying disease.

**Figure 2:**
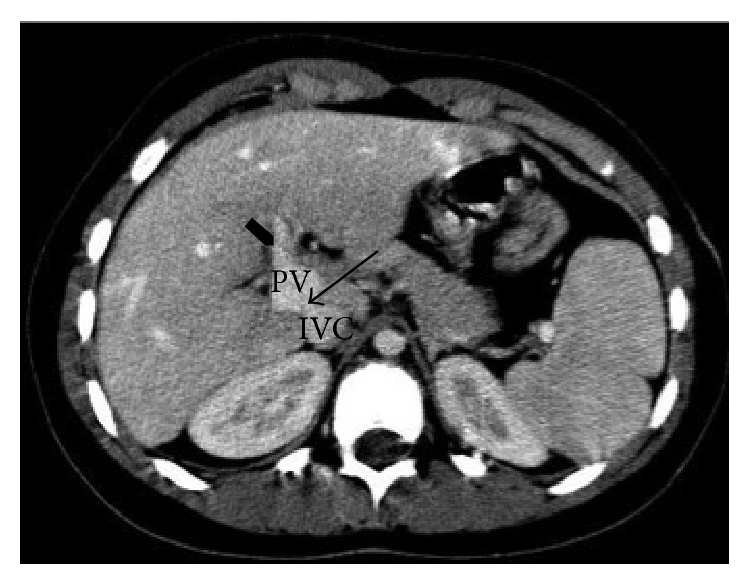
Axial contrast-enhanced CT images. The arrow shows the shunt between the portal vein (PV) and the inferior cava vein (ICV); at the hepatic hilum, the PV appears enlarged with only one intrahepatic portal branch (arrowhead).

At initial evaluation, she was in good health. Her height was 117.5 cm (<3rd percentile), her weight was 30 kg (>50th percentile), and her head circumference was 45 cm (<3rd percentile). During her hospitalization, laboratory tests were found to be unremarkable. Doppler ultrasonography was performed, revealing a slightly overflowing liver of coarse heterogeneous echotexture, with hypoechoic periportal striae. The pedicle had an irregular morphology with a hyperechoic porta and hypoechoic shadow. There was an apparent communication (10 mm) between the portal vein and inferior vena cava compatible with a portocaval shunt. Color Doppler US showed demodulated, irregular venous flow with no extra- or intrahepatic biliary ductal dilation. An abdominal CT scan was performed to study the splenic-mesenteric-portal axis. The superior mesenteric and portal vein had an enlarged caliber with slight ectasia of the celiac tripod, common hepatic artery, and its branches. The portocaval shunt had only one intrahepatic portal branch, which was of lower caliber (Figure 2). Based on these findings, a diagnosis of Abernethy malformation (Type II, side-to-side shunt) was made. As the shunt was clinically insignificant, we chose to observe the patient. To date, the patient has been followed up with periodic clinical examination and Doppler US; the patient's health has not worsened clinically.

## 4. Discussion

Congenital portosystemic shunts are classified by their anatomical characteristics into extrahepatic and intrahepatic varieties. Congenital extrahepatic portosystemic shunt (CEPS) was first described by Abernethy in 1793 [5]. In CEPS, the anastomoses are established between the portomesenteric vasculature, before division of the portal vein, and a systemic vein.

In 1994, Morgan and Superina [6] classified CEPS into two types.

- Type 1: there is complete diversion of portal blood into the systemic circulation (end-to-side shunt), with absent intrahepatic portal branches.
- Type 2: intrahepatic portal vein is intact, but some of the portal flow is diverted into a systemic vein through a side-to-side shunt.

Congenital intrahepatic shunts, first described by Raskin in 1964, are abnormal intrahepatic connections (diameter > 1 mm) between branches of the portal vein and the hepatic veins or inferior vena cava [7]. Park et al. [8] subdivided them as follows.

- Type 1: a single large vessel connecting the right portal vein to the inferior vena cava;
- Type 2: a localized peripheral shunt in which one hepatic segment has one or more communications between peripheral branches of the portal vein and the hepatic veins;
- Type 3: an aneurysmal communication between the peripheral portal vein and the hepatic veins;
- Type 4: multiple communications between the portal vein and the hepatic veins, distributed in both lobes.

Congenital portosystemic shunts can cause a broad spectrum of clinical manifestations. The liver, central nervous system, and respiratory tract are usually involved. In a high percentage of cases portosystemic shunt can lead to metabolic dysregulation, while damage to other organs is described in a very small number of cases (Table 1). Table 1 shows the principal clinical manifestations reported in the literature.

Due to the wide variability in clinical presentation, imaging plays an important role to recognize the shunt and related malformations. Color Doppler US demonstrates the presence of the shunt, the type, and the direction of flow of the identified vessels [16]. MR angiography could provide additional information about the hepatic vascular and parenchymal abnormalities. Although using methods that minimize exposure to ionizing radiation is preferable in pediatric patients, this imaging alone is insufficient. Computed tomography angiography is considered the first choice examination [17] because this method displays even small vascular branches compatible with a portocaval shunt. CT also allows imaging of intrahepatic lesions of very small dimensions, as we presented in our first case.

Therapeutic options depend on the type of shunt and its clinical course. If a Type 2 extrahepatic shunt is asymptomatic, as occurs in most children, watchful waiting is indicated and treatment recommended for the first appearance of clinical manifestations inherited to hepatic encephalopathy and liver dysfunction or complications such as pulmonary hypertension. However, these symptoms usually appear in adulthood, especially in those patients with shunts ratio above 60% [18, 19]. Therefore, meticulous clinical and sonographic monitoring must be performed. To date, there are no guidelines on the advisability of earlier treatment only to prevent complications from developing.

Treatment is indicated for patients with clinically significant shunting. Preoperatively, it is necessary to define the shunt anatomically and functionally by invasive endovascular techniques, such as catheter angiography [20]. Shunt occlusion can be performed surgically or with percutaneous endovascular procedures [16]. The aim is to occlude the shunt, while avoiding a rise in portal pressure (secondary portal hypertension). Preoperatively, it is mandatory to complete the anatomical and functional study of the shunt by more invasive techniques, such as catheter angiography [21]. In fact, in some cases, the extremely hypoplastic portal veins distal to the shunt are sometimes difficult to visualize by conventional CT angiography; in these cases, the direct catheterization of the shunt by interventional radiology is essential to study the real vascular anatomy and to distinguish the true Type 1 fistulae (absolute lack of opacification of intrahepatic portal branches) from the Type 2 fistulae with very small portal vein branches [20].

Franchi-Abella et al. [22] proposed a “balloon occlusion test” to estimate the portal pressure trend after temporary closure of the shunt: if the risk for developing portal hypertension would be insignificant, embolization would be performed; otherwise, stepwise treatment, with gradual shunt closure, would be progressively performed to acclimatize the intrahepatic portal system to the new flow, resulting from the spread of hypoplastic intrahepatic portal branches.

In patients with a Type 1 shunt, shunt occlusion is not an option, because it represents the only drainage route for mesenteric and splenic venous blood. In these cases, liver transplantation is the therapeutic approach [23].

## 5. Conclusion

Due to the wide spectrum of clinical and anatomical features, the diagnosis of portosystemic shunt can occur incidentally. Perhaps the incidence of this rare malformation is underestimated, as the disease often remains undiscovered for several years. Improved knowledge, especially about the clinical aspects, may help to lower the threshold for diagnosis. As we described in the first case, the suspicion may arise at the presence of otherwise unexplained signs and symptoms, such as occasional hypoglycemia. The second case described suggests that, in chromosomal syndromes, such as Down's syndrome, the association with this malformation needs further consideration. Finally, clinical diagnosis must be followed by radiographic evaluation, which is of primary importance to make the diagnosis and to plan management, thus avoiding the most severe consequences of this malformation.

## Figures and Tables

**Table 1:** Clinical manifestations associated with congenital portosystemic shunt [9–15].

Hepatic	Nodular lesions, focal nodular hyperplasia, hepatocellular adenoma, hepatocellular carcinoma, hepatic sarcoma, and newborn cholestasis

Neurological	Behavioral disorders, irritability, dyslexia, lethargy, EEG abnormalities, extrapyramidal signs, and epilepsy

Pulmonary	Dyspnea caused by pulmonary hypertension

Metabolic	Hyperammonemia, hypoglycemia, hyperinsulinaemia, and hypergalactosemia, without evidence of a deficiency of galactokinase or epimerase [1]

Others	IUGR, membranoproliferative glomerulonephritis with proteinuria and IgA stores, coagulation disorders, congestive heart failure, hyperandrogenism, pancreatitis, and autoimmune disorders

## Abbreviations

US: Ultrasonography
IVC: Inferior vena cava
PV: Portal vein
MRI: Magnetic resonance imaging
CT: Computed tomography
CEPS: Congenital extrahepatic portosystemic shunt.
